# The Association of TNF-Alpha Inhibitors and Development of IgA Nephropathy in Patients with Rheumatoid Arthritis and Diabetes

**DOI:** 10.1155/2020/9480860

**Published:** 2020-04-20

**Authors:** Vedran Premužić, Ivan Padjen, Mislav Cerovec, Marijana Ćorić, Bojan Jelaković, Branimir Anić

**Affiliations:** ^1^Department of Nephrology, Hypertension, Dialysis and Transplantation, University Hospital Centre Zagreb, Kispaticeva 12, Zagreb 10 000, Croatia; ^2^Department of Immunology and Rheumatology, University Hospital Centre Zagreb, Kispaticeva 12, Zagreb 10 000, Croatia; ^3^Department of Pathology, University Hospital Centre Zagreb, Kispaticeva 12, Zagreb 10 000, Croatia

## Abstract

IgA nephropathy (IgAN) is a rather uncommon complication of TNF-alpha inhibition with a range of findings such as asymptomatic microscopic/macroscopic hematuria or different degrees of proteinuria and could progress to end-stage renal disease. We are reporting three patients with longstanding rheumatoid arthritis (RA), which developed IgAN while receiving TNF-alpha inhibitors. All off our three patients had RA, which lasted 2–4 years, and none of them had a prior history of chronic kidney disease. Two patients were treated with adalimumab while one patient was treated with golimumab. Discontinuation of anti-TNF-alpha therapy and initiation of immunosuppressive therapy led to improvement in serologic abnormalities and renal function in two patients, while the third patient's 24-hour proteinuria was only partially reduced, which supports previous reports on TNF-alpha inhibitor induced autoimmunity. Two of our patients had previously been diagnosed with type 2 diabetes mellitus while the third patient developed diabetes years after the onset of IgAN. This is in line with the previously described association of IgAN and diabetes mellitus. To our best knowledge, this is the first report to analyze the development of IgAN as a potential consequence of anti-TNF-alpha therapy and its possible association with pretreatment or posttreatment diabetes.

## 1. Introduction

Introduction of TNF-alpha inhibitors has tremendously improved outcomes in patients with rheumatoid arthritis (RA). However, TNF-alpha inhibition in RA has been associated with various renal diseases including (proliferative lupus glomerulonephritis, pauci-immune necrotizing and crescentic glomerulonephritis, membranous glomerulonephritis, and renal vasculitis) [1–3]. Several centers have reported cases of IgA nephropathy (IgAN) related to treatment with TNF-alpha inhibitors [4–6]: the diagnosis of IgAN was based on typical pathohistological findings indistinguishable from idiopathic IgAN and/or nephritis associated with IgA vasculitis. Onset of IgAN associated with initiation of an anti-TNF-alpha agent and regression of renal impairment after drug withdrawal seem to be useful clues for diagnosing this entity. Current evidence on the mechanisms and predictors of development of IgAN in RA patients treated with TNF-alpha inhibitors is insufficient. The main reason is the paucity of data due to the low incidence of IgAN in the population of RA patients treated with this class of agents, as well the population of RA patients in general.

We report on three RA patients that developed IgAN during the course of treatment with TNF-alpha inhibitor.

### 1.1. Case 1

A 33-year-old man was referred to our rheumatology department in 2003 because of low back pain accompanied by tenderness of the knees and small joints of the wrists. Since the axial pattern of affection dominated the clinical presentation at that time and the patient was HLA B27 positive, he was diagnosed with ankylosing spondylitis with accompanying peripheral arthritis. Low-dose glucocorticoids and methotrexate (15 mg weekly) were introduced, leading to significant improvement of signs and symptoms, as well as decline in the number of swollen and tender peripheral joints within the following months. Basal blood pressure values were normal (128/76 mmHg) while the estimated glomerular filtration rate (eGFR) was 99.8 mL/min/1.73 m^2^.

Despite initial improvement, the following time course was marked by aggravation of signs and symptoms consistent with peripheral polyarthritis, leading to a diagnosis of seronegative RA in 2005, fulfilling the 1987 classification criteria [7]. Methotrexate was continued, now in combination with sulfasalazine (2 grams daily) being replaced with leflunomide (20 mg daily) after several months. Despite combined treatment with conventional disease-modifying agents (DMARDs) and concomitant use of low-dose glucocorticoids, the patient suffered from a persistently active disease with a 28-joint disease activity score calculated using the erythrocyte sedimentation rate, ESR (DAS28-ESR) of 5.52. This prompted the initiation of adalimumab (40 mg subcutaneous every other week), while methotrexate was continued at a lower dose (10 mg weekly). This treatment strategy led to a satisfactory clinical response and reduction of DAS28-ESR to 2.66.

In 2006, the patient developed a psoriatic rash of the palms and soles, which was successfully treated with topical therapy. In the same year, the patient developed arterial hypertension (175/94 mmHg), for which an ACE inhibitor was introduced.

In 2011, the patient was still in stabile remission of his rheumatic condition (s) while continuously taking the biological drug; however, routine urinalysis unexpectedly revealed microscopic hematuria (urine sediment E 20–30 erythrocytes and 66–73% dysmorphic erythrocytes), accompanied by non-nephrotic proteinuria (2.25 g in daily urine, dU) with eGFR of 56 mL/min/1.73 m^2^. Urine cytology revealed no urothelial atypia, and urine was negative for *M. tuberculosis*. The erythrocyte sedimentation rate (ESR) was increased (64 mm/h) as well as the C-reactive protein (CRP) level (18.3 mg/L). The complete blood count was unremarkable, as well as blood urea nitrogen (BUN) (5.0 mg/dl) and serum electrolytes. Serum creatinine was increased (137 *μ*mol/l), as well as total cholesterol (5.7 mmol/L), LDL-cholesterol (3.52 mmol/L), and triglycerides (2.78 mmol/L). Antinuclear, anti-neutrophil cytoplasmic, and anti-glomerular basement membrane antibodies (ANA, ANCA, and GBM, respectively) were negative. Serum protein electrophoresis, immunoelectrophoresis, and immunofixation were normal as well as serum complement levels (C3 and C4). Screening for hepatitis B, hepatitis C, and HIV (human immunodeficiency virus) were negative. On ultrasonography, both kidneys were of normal size with hyperechogenic parenchyma.

**Figure 1:**
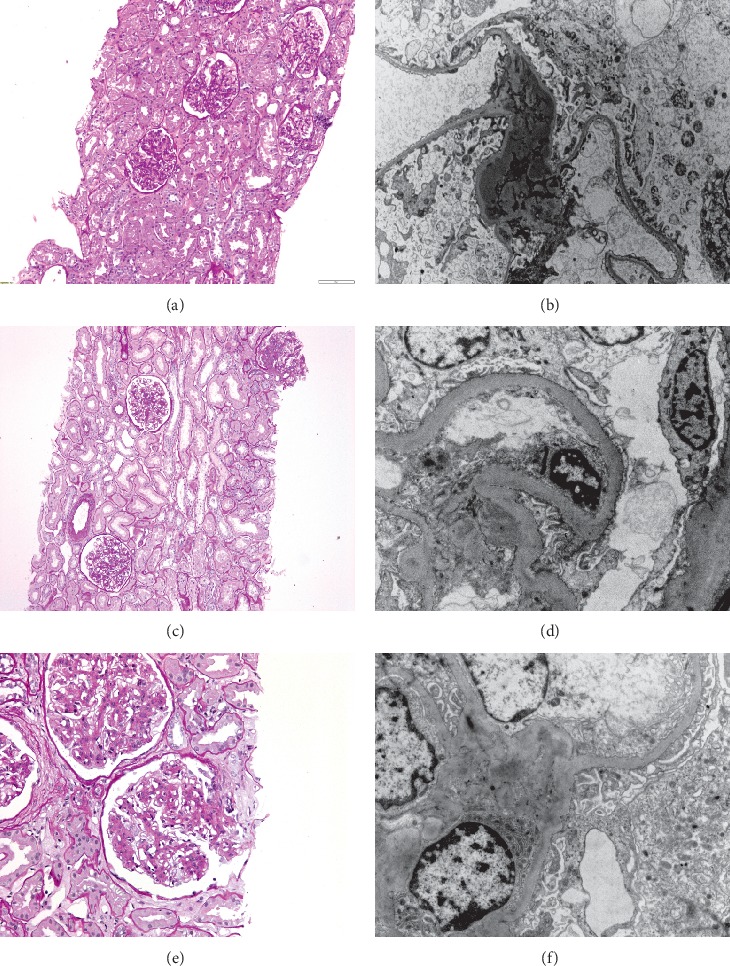
Kidney pathology of three cases. Case 1: (a) glomeruls with normal appearance (PSA stain × 100); (b) diffuse mesangial immune deposits (electron microscopy × 2800). Case 2: (c) one glomeruls with perihilar segmental sclerosis (PSA stain × 100); (d) mesangial immune deposits (electron microscopy × 5600). Case 3: (e) glomeruls with diffuse mesangial hypercellularity (PSA stain × 200); (f) mesangial immune deposits (electron microscopy × 7500).

Percutaneous kidney biopsy was performed, and its results are shown in Figures 1(a) and 1(b).

On light microscopy, diffuse mesangial hypercellularity was observed in all glomeruli, whereas global sclerosis was established in 1 of 15 glomeruli (6.66%). The tubules demonstrated degenerative changes while there were no changes on arteries. A moderate mononuclear infiltrate and mild fibrosis was noted in the interstitium. Immunofluorescence microscopy revealed mild mesangioproliferative changes. Electronic microscopy showed eosinophil infiltrates mostly in the mesangium, while several subendothelial and subepithelial eosinophil deposits were seen in the capillaries. In some glomeruli, there were signs of podocyte loss. Electronic microscopy also revealed glomerulonephritis with diffuse mesangial deposits and several subendothelial and subepithelial deposits compliant with glomerulonephritis caused by circulating immune complexes. The findings were in concert with the diagnosis of IgAN. There were no elements of diabetic nephropathy. The patient's MEST-C score was M1, E0, S1, T2, and C0.

Treatment with adalimumab and medium-dose glucocorticoids (prednisone 20 mg) was continued as well as an ACE inhibitor. Over the next two years, the patient was in a state of low disease activity (DAS28 3.08) of his RA, with progression of chronic kidney disease (eGFR 35.3 mL/min/1.73 m^2^) and persistent proteinuria (2.26 g/dU). This prompted discontinuation of adalimumab and a further increase in the dose of glucocorticoids (prednisone 60 mg), with the intention to control the renal disease.

Due to the increased prednisone dose, the patient gradually developed cushingoid features including type 2 diabetes mellitus; he was diagnosed in 2014. A slight improvement in kidney function (eGFR 44.1 mL/min/1.73 m^2^) and proteinuria (1.60 g/dU) allowed for a reduction of the prednisone dose to 40 mg. Of note, the patient's blood pressure was unremarkable, often being 120/80 mmHg or even less.

In 2015, the patient's kidney function was without further deterioration (eGFR 42.2 mL/min/1.73 m^2^) and without progression of proteinuria. Diabetes was not adequately controlled, and the patient also experienced a relapse of his RA; so, rituximab was introduced in 2016. Unfortunately, the drug had to be discontinued after three cycles due to lacking efficacy and an increase in proteinuria from 0.78 to 2.04 g/dU. After discontinuation of rituximab, a decision was made to commence treatment with baricitinib, an oral selective JAK inhibitor. This finally led to the achievement of a low disease activity state of RA. However, the patient still continued to have persistent proteinuria of 1.9 g/dU, with no further decline in the eGFR.

### 1.2. Case 2

A 27-year-old woman was referred in 2007 to our rheumatology department because she had tenderness of her feet and knees with normal levels of acute phase parameters. Nine years before this referral, she was diagnosed with diabetes type 1 with consequent mild retinopathy and neuropathy.

Given that she had no swollen joint, an expectative approach was initially taken, and she was treated with NSAIDs. However, in early 2009, she developed symmetrical peripheral polyarthritis consistent with a diagnosis of seronegative RA, and she was put on low-dose methylprednisolone (8 mg qd) and weekly methotrexate (15 mg). Due to suboptimal response, sulfasalazine (2 grams daily) was added to the aforementioned regimen. Her blood pressure values were normal (122/64 mmHg), while her estimated glomerular filtration rate (eGFR) was 103.2 mL/min/1.73 m^2^. Although low disease activity was achieved in the following months, she again started to develop active disease with swollen and tender joints of her hands, feet, and knees. A pattern of persistently active disease with a DAS28-ESR exceeding 5.00 led to the decision to start the patient on golimumab (50 mg subcutaneously, once monthly) in 2012. Achievement of sustained clinical remission allowed the discontinuation of glucocorticoids, while the dose of methotrexate was also gradually tapered, and the drug was finally stopped in 2013. Around that time, the patient developed arterial hypertension (160/90 mmHg), with an intact renal function.

In 2015, the patient was still in persistent remission of her rheumatic disease. Routine screening of her 24 h urine revealed albuminuria of 420.53 mg/dU and proteinuria of 0.47 g/dU. The findings were accompanied by a history of oscillatory hypertension and occasional ankle oedema. Laboratory tests revealed the following: hemoglobin: 11.7 g/dl, hematocrit: 37%, white blood cell count: 7.1 × 10^9^/L, platelet count: 247 × 10^9^/L, BUN: 4.9 mg/dl, serum creatinine: 61 µmol/L, eGFR 112.8 mL/min/1.73 m^2^, albumin: 34.6 g/L, cholesterol: 4.6 mmol/L, triglycerides 0.61 mmol/L, and LDL-cholesterol: 2.36 mmol/L. Serum electrolytes were within normal limits. The erythrocyte sedimentation rate (ESR) was 20 mm/h, while the level of C-reactive protein (CRP) was normal. Serum protein electrophoresis and classes of immunoglobulins (IgG, IgA, and IgM) were within normal limits. Immunological screening (comprised of complement components C3 and C4, anti-neutrophil cytoplasmic antibodies, antinuclear antibodies including extractable nuclear antigens, and anti-glomerular basement membrane antibodies) was also unremarkable. Screening for hepatitis B and C, as well as HIV) was negative. On ultrasonography, both kidneys were of normal size, with a hyperechogenic parenchyma.

Percutaneous kidney biopsy was performed, and the pathohistological findings are shown in Figures 1(c) and 1(d).

On light microscopy, diffuse mesangial hypercellularity was demonstrated in all glomeruli, whereas a perihilar segment of sclerosis was established in 1 of 17 glomeruli (5.88%). Degenerative changes were seen in the tubules, while segmental intimal deposits were described in the arteries. There were no signs suggestive of interstitial inflammation and/or fibrosis.

Immunofluorescence microscopy showed diffuse IgA, moderate C3, and lambda light chains, and low IgM and kappa light chains deposits in the mesangium while there were no IgG, C1q, and fibrin deposits. Moderate C3 deposits were described in the arteries. Electronic microscopy revealed eosinophil infiltrates mostly in the mesangium and several subendothelial and subepithelial eosinophil deposits in the capillaries. Loss of podocytes was described in some of the glomeruli. There were no elements of diabetic nephropathy. The biopsy findings were consistent with IgAN class II, with a MEST-C score being M1, E1, S1, T2, and C0.

Given the remarkable and sustained response of RA to golimumab, the biological drug was continued, with the addition of an ACE inhibitor. During 2016, the patient was still in clinical remission of her RA with a stabile kidney function (eGFR 60.2 mL/min/1.73 m^2^), but proteinuria increased to 3.0 g/dU. This led to the decision to discontinue golimumab and start the patient on oral cyclophosphamide (50 mg bid) with 40 mg of prednisone. Three months later after introduction of Endoxan, proteinuria dropped to 1.03 g/dU (partial remission); thus, cyclophosphamide was replaced with azathioprine (50 mg bid), with gradual tapering of prednisone. It is noteworthy that the kidney function did not decline over the follow-up period (eGFR 62.5 mL/min/1.73 m^2^), while proteinuria continued to gradually improve (to 0.65 g/dU), despite inadequately controlled glycemia (provoked due to relatively high doses of prednisone). Such an improvement allowed for a further tapering of prednisone. In the following year, rituximab was introduced as a biological disease-modifying agent to control rheumatoid arthritis (1 gram on days 0 and 14, repeated every 6 months). Given that it was found reasonable to expect that rituximab may serve as a sound option for the maintenance of remission of the patient's renal disease, azathioprine was discontinued. In the following course (after three cycles of rituximab), the patient continued to be in clinical remission of her RA, but also achieved a complete decline in proteinuria to 0.09 g/dU with normal kidney function (eGFR was 73.3 mL/min/1.73 m^2^) and good blood pressure control (<130/90 mmHg).

### 1.3. Case 3

A 48-year-old woman was referred to our rheumatology department in 2004 due to tenderness of her small joints of the wrists and knees, accompanied by morning stiffness of the lower back. Four years prior to the first visit, she was diagnosed with diabetes type 2. After initial workup, she was diagnosed as undifferentiated polyarthritis and was treated with NSAIDs and medium- to low-dose prednisone with good response and relief of symptoms. In 2009, she was finally diagnosed as seropositive rheumatoid arthritis with high titers of both RF and anti-CCP antibodies. She was put on 15 mg of methotrexate weekly, as well as sulfasalazine 2 grams daily.

In 2011, RA was still clinically active with increased inflammatory parameters and high DAS28-ESR of 7.09, so methotrexate was increased to 25 mg once a week but without considerable clinical effect. Therefore, a TNF-*α* inhibitor was introduced; she was started on adalimumab (40 mg subcutaneous every other week) with methotrexate 25 mg once weekly. She continued to take low-dose prednisone (up to 7.5 mg daily), achieving a satisfactory clinical response and reduction of DAS28-ESR to 2.28.

However, in 2015, she again started developing clinically active disease, but with normal inflammatory parameters. In 2016, she developed increased inflammatory markers with development of puffy pretibial edema and microcytic anemia. Laboratory tests showed hemoglobin: 10.5 g/dl, hematocrit: 33%, mean corpuscular volume: 73.2 fL, white blood cell count: 13.3 × 10^9^/L, platelet count: 297 × 10^9^/L, BUN: 8.5 mg/dl, serum creatinine: 81 µmol/l, eGFR: 68 mL/min/1.73 m^2^, albumin: 31.0 g/L, cholesterol: 5.6 mmol/L, triglyceride: 1.65 mmol/L, and LDL-cholesterol: 3.59 mmol/L. Serum electrolytes were within normal limits. Proteinuria was 4.55 g/dU. Erythrocyte sedimentation rate (ESR) was 100 mm/h, CRP 45.9 mg/L. Serum complement levels (C3 and C4), ANA, ANCA, and anti-glomerular basement membrane (GBM) antibodies were all unremarkable. Serum protein electrophoresis revealed polyclonal hypergammaglobulinemia while immunofixation was normal. Screening for hepatitis B, hepatitis C, and HIV was negative. On ultrasonography, both kidneys were of normal size, with hyperechogenic parenchyma.

Percutaneous kidney biopsy was performed; results of the histopathological analyses are shown in Figures 1(e) and 1(f).

Light microscopy revealed diffuse mesangial hypercellularity with segmental formation of nodules in all glomeruli, whereas global sclerosis was established in 5 of 22 glomeruli (22.7%). Degenerative changes were demonstrated in the tubules, while the findings in the arterioles included concentric intimal hyalinosis and moderate intimal fibrosis with elastosis. There was no mononuclear infiltrate but fibrosis was present in interstitium.

Immunofluorescence microscopy showed diffuse IgA, moderate C3 and lambda light chains, and low IgM and kappa light chain deposits in the mesangium while there were no IgG and C1q deposits. Moderate C3 deposits were detected in the arteries. Electronic microscopy showed osmiophilic deposits mostly in the mesangium and paramesangium deposits. Glomerular basal membranes were a bit thicker without osmiophilic deposits, with stationary thicker internal lamina rara. Podocyte loss was described in some of the glomeruli. Therefore, a diagnosis of diabetic nephropathy with superimposed IgAN was established, prompting the decision to discontinue adalimumab. The patient's MEST-C score was M1, E1, S1, T2, and C0. The cause of microcytic anemia was two polyps of the colon which were removed. At that time, the patient developed arterial hypertension (170/90 mmHg) and an ACE inhibitor was introduced, with the aim to control hypertension and to control the proteinuria. Arthritis was suboptimally controlled with low-dose steroids. The patient continued to have nephrotic-range proteinuria, as well as high disease activity of her RA. This prompted the decision to target both patient's conditions with a single agent, rituximab, administered in 4 cycles. This led to remission of arthritis, as well as a pronounced decline in proteinuria to 0.13 g/dU, with a stabile kidney function, which was sustained through the following period (eGFR 65 mL/min/1.73 m^2^).

## 2. Discussion

TNF-alpha inhibition in patients with RA may lead to the development of new-onset renal disease [4, 8]. IgAN is a rather uncommon complication of TNF-alpha inhibition with a range of findings such as asymptomatic microscopic/macroscopic hematuria or different degrees of proteinuria and could progress to end-stage renal disease [8, 9]. Renal biopsy reveals classical features with or without C3 activation, which discriminates classic from crescentic IgAN. It is worth noting that neither the complement components nor immunoglobulins were detected in kidney biopsies of our patients.

The exact pathophysiological mechanism for the development of IgAN in patients treated with TNF-alpha inhibitors has not yet been determined. Anti-TNF-alpha agents may lead to autoantibody formation including antinuclear, anti-double stranded DNA (anti-dsDNA), and anticardiolipin antibodies [10, 11]. Binding of anti-TNF-alpha on immune cells could induce a release of immunogenic nucleosomal antigens, which promote the production of anti-dsDNA antibodies. Recently, a correlation between elevated concentrations of circulating TNF-alpha and renal progression in IgAN has been reported [12]. The activation of alternative complement pathway including C3 and formation of deposits could be responsible for IgAN development. Therefore, biological therapy could induce an immune response with the production of IgA and IgM antibodies against TNF-alpha inhibitors.

**Figure 2:**
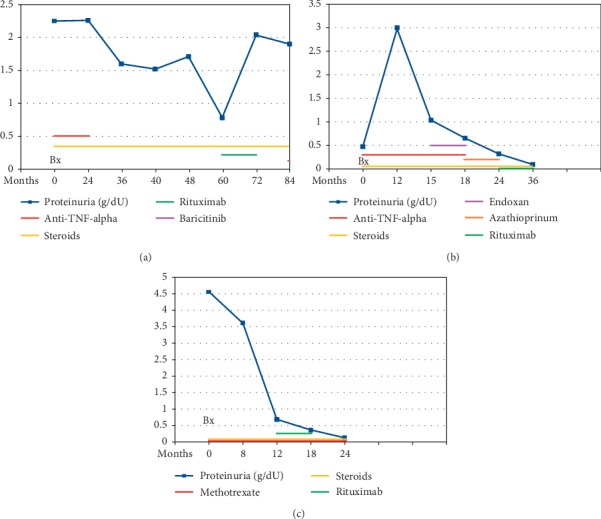
Proteinuria and therapy after kidney biopsy. Case 1 (a); Case 2 (b); Case 3 (c).

We are reporting three patients with longstanding RA, which developed IgAN while receiving TNF-alpha inhibitors. All of our three patients had RA, which lasted 2–4 years, and none of them had a prior history of chronic kidney disease (Table 1). Two patients were treated with adalimumab, while one patient was treated with golimumab (treatment duration: 36–70 months, median: 55 months). The development of IgAN occurred after months of therapy and included pathologic and serologic findings specific for the pathogenic role of TNF-alpha inhibitors. Discontinuation of anti-TNF-alpha therapy and initiation of immunosuppressive therapy led to improvement in serologic abnormalities and renal function in two patients, while the third patient's 24-hour proteinuria was only partially reduced (Table 2 and Figures 2(a)–2(c)), which supports previous reports on TNF-alpha inhibitor induced autoimmunity. Since all of our patients developed IgAN while on TNF-alpha inhibiting treatment, it is plausible to assume that IgAN developed as a consequence of the biological treatment. It is less likely that IgAN was triggered by rheumatoid arthritis, given that the rheumatic disease was adequately controlled (i.e. in remission or low disease activity) following the institution of a TNF-alpha inhibiting drug. Furthermore, none of the presented patients exhibited features of renal affection prior to the institution of the respective TNF-alpha inhibitor. Although cases of IgAN were reported in groups of patients with RA, it is worth noting that a cause-effect relationship between RA and IgAN has never been confirmed and that it is likely that these observations were coincidental. Moreover, the finding of a relatively high percentage (12%) of IgAN in a Japanese group of 100 patients has not been replicated ever since [13]. This observation should be interpreted with caution because the patients included in the respective study originate from an era when RA had been commonly recognized at a more established stage. Additionally, therapeutic approaches used at the time when the study was conducted did not meet today's stringent standards and aims.

Interestingly, two of our patients had previously been diagnosed with type 2 diabetes mellitus, while the third patient developed diabetes years after the onset of IgAN. This is in line with the previously described association of IgAN and diabetes mellitus [14]. Given that type 2 diabetes mellitus has already been associated with other immune-mediated entities such as celiac disease and dermatitis herpetiformis, it may be reasonable to suggest that the occurrence of IgAN in diabetic patients is not a mere coincidence. Some studies reported that patients with diabetes had higher values of IgA when compared to healthy controls [14, 15]. This may be a consequence of enhanced antigen stimulation as an immune response to advanced glycosylation end products and diabetic metabolic disturbance, finally leading to nonenzymatic glycation of immunoglobulins, especially IgA.

IgA levels are further increased in patients with IgAN superimposed to diabetes. To our best knowledge, this is the first report to analyze the development of IgAN as a potential consequence of anti-TNF-alpha therapy and its possible association with pretreatment or posttreatment diabetes. Taking into account already elevated IgA levels in diabetic patients, the administration of biologic treatment could hasten the development of IgAN, although TNF-alpha inhibitors are of IgG class. A strategy of biologic drug discontinuation combined with initiation of an immunosuppressive drug led to IgAN remission in two patients. The third patient still had significant proteinuria and stationary chronic kidney disease, although he had received two biologic drugs. A possible explanation could be the development of diabetes after initial biologic treatment and persistently elevated IgA levels. Switching to the patient's current treatment with baricitinib (a Janus kinase inhibitor) unfortunately did not yield a significant reduction in proteinuria. Combining the MEST-C score with clinical data at biopsy has the same predictive value as monitoring clinical findings for two years [16]. The score takes into account four features: mesangial and endocapillary cellularity, segmental sclerosis, and interstitial fibrosis/tubular atrophy. The scoring of each biopsy sample (for instance M1, E0, S1, and T1) gives us a predicted rate of decline in renal function over a follow-up period of 59 months where 12% patients progressed to ESRD. Two of our patients did not have a decline in renal function despite their MEST-C score being M1, E0, S1, T2, and C0. The third patient had a reduced glomerular filtration rate but without progression in a four-year follow-up period. Our findings confirmed previous reports on the development of IgAN after biologic treatment with TNF-alpha inhibitors. Although this is a report on only three patients, it could be speculated that the presence of diabetes may act as a risk factor for the development of IgAN in patients with RA treated with TNF-alpha inhibitors. Determining serum IgA levels could have a prognostic importance in predicting the development of IgA nephropathy superimposed on diabetes mellitus in patients with RA. We suggest that RA patients, especially those with high risk of developing renal disorders should be prescribed TNF-alpha inhibitors cautiously and should be closely monitored over an extended period of time.

## Figures and Tables

**Table 1:** Clinical characteristics of three RA patients with IgA glomerulonephritis following anti-TNF-*α* therapy.

	Case 1	Case 2	Case 3
Months from first visit to diagnosis of RA	22	24	57
Steroids in months	22	5	51
Methotrexate in months	3	36	23
Prior diabetes	No	Yes	Yes
Prior arterial hypertension	No	No	No

Months from diagnosis of RA to introduction of biologic treatment	48	34	27
DAS28 at biologic treatment introduction	5.52	6.30	7.09
DAS28 after biologic treatment introduction	2.66	2.09	2.28
TNF-*α* inhibitor	Adalimumab	Golimumab	Adalimumab

Months from introduction of biologic treatment to kidney biopsy	59	36	70
Peripheral oedema	No	Yes	Yes
24 h urine proteinuria (g/dU)	2.25	0.47	4.55
Erithrocyturia	Yes	No	No
Serum creatinine (*μ*mol/L)	137	61	81
eGFR (mL/min/1.73 m^2^)	54.8	112.8	68.0
Blood pressure (mmHg)	175/94	160/90	170/90
Arterial hypertension	Yes	Yes	Yes
Hemoglobin (g/dl)	13.1	11.7	10.5
White blood cell count (×10^9^/L)	7.8	7.1	13.3
Cholesterol (mmol/L)	5.7	4.6	5.6
Triglycerides (mmol/L)	2.78	0.61	1.65
Erythrocyte sedimentation rate (mm/h)	64	20	100
C-reactive protein (mg/L)	18.3	1.7	45.9

RA-rheumatoid arthritis; eGFR-estimated glomerular filtration rate.

**Table 2:** Clinical characteristics and renal survival of three RA patients with IgA glomerulonephritis following kidney biopsy.

	Case 1	Case 2	Case 3
METS-C score	M1, E0, S1, T2, C0	M1, E1, S1, T2, C0	M1, E1, S1, T2, C0
*Therapy after kidney biopsy*			
Steroids in months	24	24	24
Methotrexate in months	—	—	24
Original TNF-*α* inhibitor in months	24	19	—
Rituximab (number of cycles)	3	3	4
Endoxan/Azathioprine	No	Yes	No
DAS28 one year after biopsy	3.08	2.76	4.54

Renal survival after biopsy	2 years	1 year	1 year
24 h urine proteinuria (g/dU)	2.26	3.00	0.68
Serum creatinine (*μ*mol/L)	195	103	88
eGFR (mL/min/1.73 m^2^)	35.3	60.2	61.1

Renal survival after biopsy	4 years	2 years	2 years
24 h urine proteinuria (g/dU)	1.57	0.09	0.13
Serum creatinine (*μ*mol/L)	166	87	83
eGFR (mL/min/1.73 m^2^)	42.2	73.3	65.1

eGFR-estimated glomerular filtration rate.
