# Corrigendum to “The Association of TNF-Alpha Inhibitors and Development of IgA Nephropathy in Patients with Rheumatoid Arthritis and Diabetes”

**DOI:** 10.1155/2020/7293470

**Published:** 2020-08-29

**Authors:** Vedran Premužić, Ivan Padjen, Mislav Cerovec, Mario Laganović, Tajana Željković-Vrkić, Jelena Kos, Marijana Ćorić, Bojan Jelaković, Branimir Anić

**Affiliations:** ^1^Department of Nephrology, Hypertension, Dialysis and Transplantation, University Hospital Centre Zagreb, Kispaticeva 12, Zagreb 10000, Croatia; ^2^Department of Immunology and Rheumatology, University Hospital Centre Zagreb, Kispaticeva 12, Zagreb 10000, Croatia; ^3^Department of Pathology, University Hospital Centre Zagreb, Kispaticeva 12, Zagreb 10000, Croatia

In the article titled “The Association of TNF-Alpha Inhibitors and Development of IgA Nephropathy in Patients with Rheumatoid Arthritis and Diabetes” [1], Dr. Mario Laganović, Dr. Tajana Željković-Vrkić, and Dr. Jelena Kos were missing from the authors' list. The authors contributed towards obtaining, analysing, and interpreting the data presented in the study. The corrected author list is shown above.
